# Similar Evolution of Cellular HIV-1 DNA Level in Darunavir/Ritonavir Monotherapy versus Triple Therapy in MONOI –ANRS136 Trial over 96 Weeks

**DOI:** 10.1371/journal.pone.0041390

**Published:** 2012-07-25

**Authors:** Sidonie Lambert-Niclot, Philippe Flandre, Marc-Antoine Valantin, Cathia Soulie, Slim Fourati, Marc Wirden, Sophie Sayon, Sophie Pakianather, Laurence Bocket, Bernard Masquelier, Georges Dos Santos, Christine Katlama, Vincent Calvez, Anne-Genevieve Marcelin

**Affiliations:** 1 AP-HP, Pitié-Salpêtrière Hospital, INSERM U 943 and Pierre et Marie Curie University Paris, Paris, France; 2 INSERM U 943, Paris, France; 3 CHRU de Lille, Virology, Lille, France; 4 AP-HP, Pellegrin Hospital, Bordeaux, France; 5 Centre Hospitalier et Universitaire de Fort-de-France, Fort de France, Martinique, France; McGill University AIDS Centre, Canada

## Abstract

**Background:**

A higher proportion of intermittent viremia (to have a HIV-1 RNA viral load>50 copies/mL not confirmed) was reported in the boosted protease inhibitor monotherapy arm in some studies including MONOI trial, and that could have an impact on the replenishment of the HIV-1 DNA reservoirs. The HIV-1 DNA level is an interesting marker which reflects the size of cellular HIV reservoir. Our objectives were to study the impact of 96 weeks of Darunavir/ritonavir monotherapy versus a triple standard combination on the HIV-1 blood reservoir and factors associated with HIV-1 plasma DNA at baseline in MONOI trial sub-study.

**Methodology/Principal Findings:**

This sub-study is focused on 160 patients (79 patients in monotherapy arm and 81 in tritherapy arm) for whom blood cells were available both at baseline and at week 96 (W96). Baseline HIV-1 plasma DNA was associated with CD4 nadir, pre therapeutic HIV-1 RNA viral load and baseline HIV-1 RNA measured by ultrasensitive assay. A similar median delta HIV-DNA was observed between D0 and W96 in both arms; 0.35 log copies/10^6^ leucocytes in monotherapy arm versus 0.51 log copies/10^6^ leucocytes in tritherapy arm.

**Conclusion/Significance:**

Despite a higher proportion of intermittent viremia in monotherapy arm, a similar evolution of cellular HIV-1 DNA level was observed between mono and triple therapy arm.

**Trial Registration:**

ClinicalTrials. gov NCT00421551 <NCT00421551>

## Introduction

Different strategies of boosted protease-inhibitor (PI) monotherapy have been evaluated [1], [2], [3] to avoid nucleosides long term toxicities and to decrease cost of treatment. In this context, MONOI trial was designed to evaluate whether darunavir/ritonavir (DRV/r) monotherapy could maintain viral suppression showing non-inferior efficacy, compared with a triple therapy arm of two nucleoside analogues plus DRV/r [4], [5]. Recently published European HIV treatment guidelines have introduced the option for a switch to PI monotherapy (either with DRV/r once daily or lopinavir/ritonavir twice daily) for patients who have HIV-RNA levels sustained <50 copies/mL and no history of virological failure (VF) [6]. However these studies reported a higher proportion of intermittent viremia (to have a viral load>50 copies/mL not confirmed) in the monotherapy arm [1], [2], [3], [4], [7] that could have an impact on the replenishment of the HIV reservoirs.

One concern was, despite maintenance of viral suppression in plasma, the use of one drug compared to three drugs could lead to an increase in viral reservoirs including body compartments and target cells. The HIV-1 DNA level is an interesting marker which reflects the size of cellular HIV reservoir. Few studies have already addressed the HIV-1 DNA decrease in chronically infected patients receiving standard triple antiretroviral therapies [8], [9]. Finally, no data are available regarding experienced patients receiving DRV/r monotherapy.

Monark study [10] had showed that non responders had significantly higher baseline HIV-1 DNA than responders in induction lopinavir/r monotherapy arm. In the MONOI study, factors associated with VF in patients receiving DRV/r monotherapy were having an initial blip, shorter time of ARV treatment before monotherapy, an adherence <100% during monotherapy and higher HIV-1 DNA level at baseline [11]. Indeed, a low HIV-1 plasma DNA level, reflecting HIV-1 reservoir, could be related to a strongest control of residual viremia.

Our objectives here were to study the impact of 96 weeks of DRV/r monotherapy versus a triple standard combination on the HIV-1 blood reservoir and factors associated with HIV-1 plasma DNA at baseline in MONOI trial sub-study.

## Methods

### Participants

**Figure 1:**
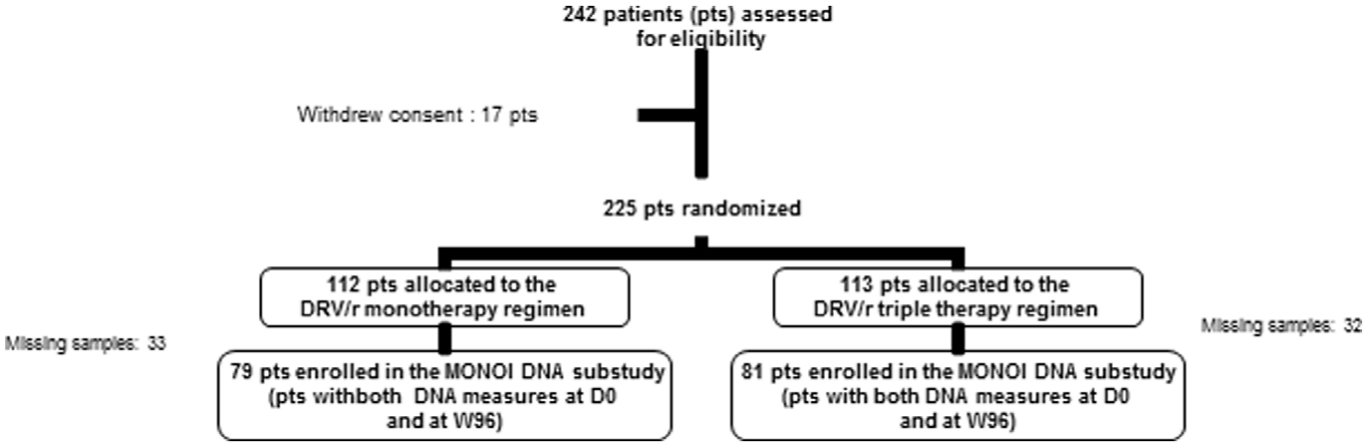
Flow chart.

Overall 225 HIV-1 infected experienced patients were randomized in the MONOI trial (113 in the triple therapy arm and 112 in the monotherapy arm). This sub-study is focused on 160 patients (79 patients in monotherapy arm and 81 in tritherapy arm) for whom blood cells were available both at baseline and at week 96 (W96) (figure 1).

The MONOI study comprised a first phase where darunavir/ritonavir 600/100 mg bid was introduced for eight weeks as a component of a triple-drug regimen in replacement of the PI, non nucleoside reverse transcriptase inhibitors (NNRTI) or third nucleoside RTI (NRTI). Patients whose HIV-1 VL remained lower than 50 copies/mL four weeks after darunavir induction were randomly assigned 1∶1 at day 0 (D0) to continue the triple drug darunavir-containing regimen (darunavir triple therapy) or to stop the two NRTIs (darunavir monotherapy). After screening (W-10), study evaluations were completed at W-4, randomization and at weeks 4, 8 and every 8 weeks thereafter for the duration of the study. The study population consisted of HIV-1 infected patients at least 18 years of age receiving a triple antiretroviral drug regimen. All patients had plasma HIV-1 RNA less than 400 copies/mL for the last eighteen months, based on at least four viral load measurements, and less than 50 copies/mL at screening. Patients had no history of virologic failure while receiving a PI containing regimen, a documented CD4 lymphocytes nadir greater than 50 cells/mm^3^, and acceptable laboratory results at screening. Patients with an history of HIVrelated neurological disease or with hepatitis B co-infection could not be enrolled.

### Ethics

The MONOI study was designed and conducted by members of the Agence Nationale de Recherche sur le SIDA et les Hépatites Virales, Paris, France (MONOI ANRS 136 trial). The protocol was approved by the ethics committee CPP Paris VI Pitié-Salpêtrière and by the Agence Française de Sécurité Sanitaire des Produits de Santé (January 23, 2007Clinical trial registration. NCT00421551). All patients provided written informed consent.

### Description of Procedures

Residual plasma viremia was measured as previously described [12] with a limit of quantification of 1 copy/ml at W-10 and Day 0 (D0). According to the baseline ultrasensitive VL results, we defined 2 groups: W-10 and D0 values of ultrasensitive VL <1 copy/mL and others. Cellular HIV-1 DNA was quantified at D0 and at W96 as previously described [13]. The study definition for VF was: two plasma HIV-1 viral load >400 copies/mL on two weeks apart. We investigated whether some variables (age, sex, CD4 nadir, duration of HIV-infection, duration of previous NRTI, NNRTI, and PI exposure, pre therapeutic VL, baseline HIV-1 RNA measured by ultrasensitive assay, BMI) were associated with log_10_ HIV-1 DNA at D0. Delta HIV DNA is the change in log10 HIV-1 DNA from D0 to W96 (log copies/10^6^ leucocytes).

### Statistical Methods

Between-group comparisons were carried out by the Kruskal-Wallis test for continuous variables and by the Fisher exact test for categorical variables. Pearson and Spearman correlation measure were used to quantify the association between log_10_ HIV-1 DNA at D0 and some continuous variables (age, CD4 nadir, duration of HIV-infection, duration of previous NRTI, NNRTI, and PI exposure, pre therapeutic VL, BMI). The Pearson coefficient was used when the variance was constant over the errors (homoscedasticity) and the Spearman was used for variance not constant (heteroscedasticity). We investigated whether sex and baseline HIV-1 RNA measured by ultrasensitive assay (2 measures below 1 copy/ml vs. other) were associated with log_10_ HIV-1 DNA at D0 by the Kruskall-Wallis test. We also investigated whether change in log_10_ HIV-1 DNA from D0 to W96 was associated with randomized groups, VF and having two consecutive HIV-1 RNA plasma viral load>50 copies/ml by Kruskall-Wallis test. There were adequate number of participants in order to determine the magnitude of the outcome measures.

## Results

**Figure 2:**
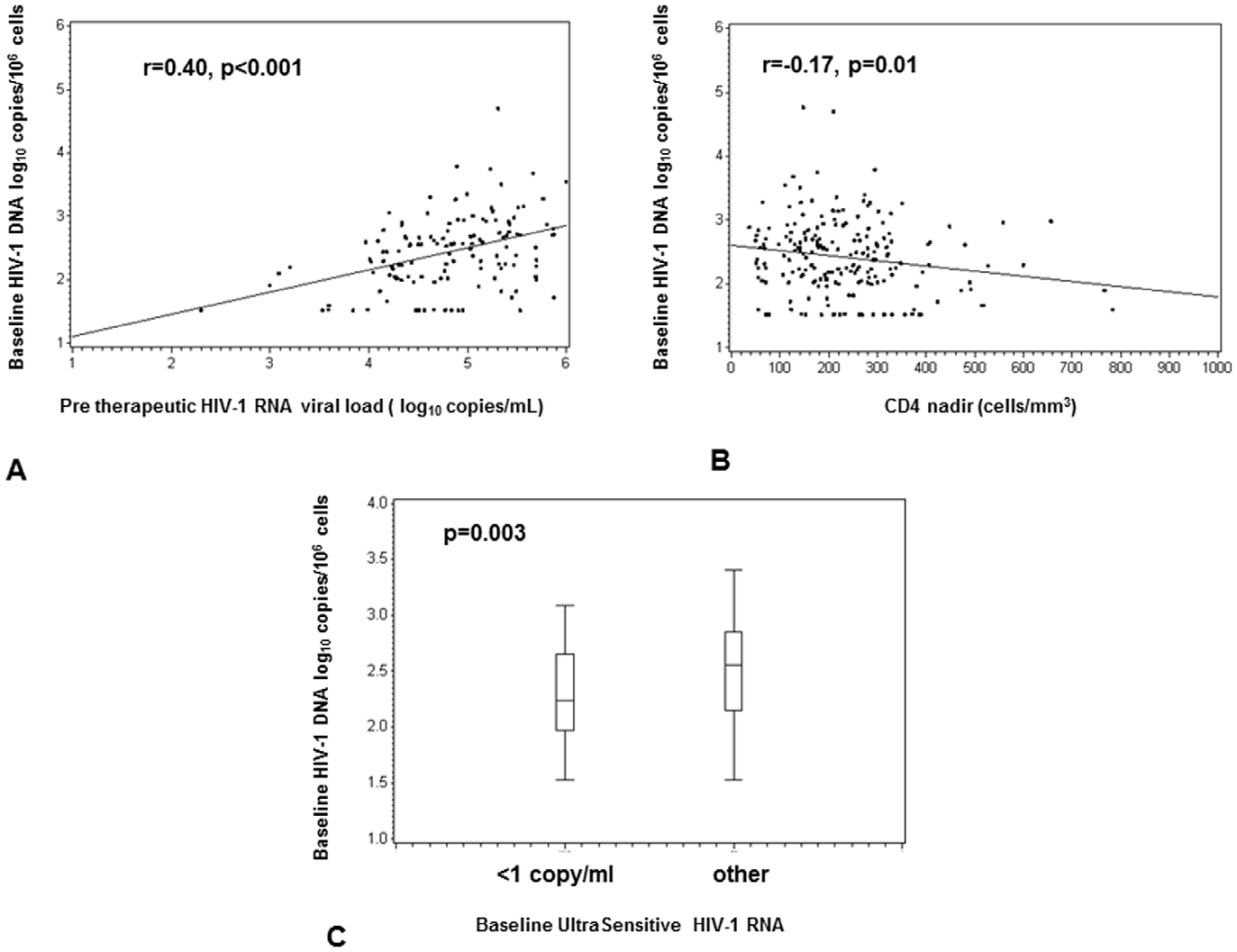
Factors associated with log_10_ HIV-DNA at baseline. A. pre therapeutic VL (Pearson test); B. CD4 nadir (Spearman test); C. baseline HIV-1 RNA measured by ultrasensitive assay (Kruskal Wallis test).

The patient’s characteristics are presented in table 1 and are not significantly different between monotherapy and tritherapy arms. These characteristics are similar to those of all patients included in MONOI (n = 225)(all P-values >0.09) [4]. The difference of proportion of intermittent viremia between triple therapy arm and monotherapy arm was not significant (32% versus 46% respectively). The percentages of undetectable HIV-1 DNA viral load (<1.53 log10 copies/10^6^ cellules) were 8% at baseline and 12% at W96, which is in the same order than previously described in patients after long term suppressive ARV therapy [14]. There was no significant difference of proportion of undetectable HIV-1 DNA viral load between monotherapy arm and tritherapy arm. A similar median delta HIV-DNA was observed between D0 and W96 in both arms; 0.35 log copies/10^6^ leucocytes in monotherapy arm versus 0.51 log copies/10^6^ leucocytes in tritherapy arm (p = 0.22). The delta HIV-1 DNA viral load was not associated with VF (2 plasma HIV-1 viral load >400 copies/mL on two weeks apart) or to have two consecutive HIV-1 RNA plasma viral load>50 copies/ml. The evolution of HIV-1 DNA viral load was not associated with intermittent viremia and to have and baseline HIV-1 RNA measured by ultrasensitive assay. Factors associated with log_10_ HIV-DNA at D0 were CD4 nadir (Spearman Test, p = 0.001), pre therapeutic VL (Pearson test, p<0.001) and baseline HIV-1 RNA measured by ultrasensitive assay (Kruskal Wallis test, p = 0.003) (Figure 2). Factor associated with baseline HIV-1 RNA measured by ultrasensitive assay was only log_10_ HIV-DNA at D0.

**Table 1:** Characteristics of patients.

	Darunavir/r triple therapy N = 81	Darunavir/r monotherapy N = 79
Age-year		
Median (IQR)	46 (40–56)	46 (41–51)
Male sex - n (%)	62 (77)	60 (76)
CD4 cells per mm3 - median (IQR)		
Baseline	591 (393–859)	582 (456–727)
Nadir	208 (144–280)	219 (144–319)
3 classes experience - n (%)	33 (41)	29 (37)
Duration of HIV infection - year		
Median (IQR)	8.1 (4.1–16.3)	11.1 (6.4–16.0)
Duration of ART - year		
Median (IQR)	7.3 (2.8–11.2)	8.2 (4.5–10.7)
Prior PI exposure - n (%)	73 (90)	64 (81)
Regimen at screening - n (%)		
2 NRTIs + PI	64 (79)	50(63)
2 NRTIs + NNRTI	11 (14)	16 (20)
3 NRTIs	5 (6)	12 (15)
Baseline UltraSensitive HIV-1 RNA – n (%)		
<1 copy/ml	30 (37%)	38 (49%)
Other	51 (63%)	40 (51%)
Baseline log_10_ DNA/10^6^ cellules		
Median (IQR)	2.45 (2.1–2.8)	2.38 (2.1–2.8)
Patient presenting Intermittent viremia - n (%)	26 (32)	36 (46)

IQR: interquartil range; ART: antiviral therapy; PI: protease inhibitor; NRTI: nucleoside reverse transcriptase inhibitor; NNRTI: non nucleoside reverse transcriptase inhibitor.

## Discussion

A higher proportion of intermittent viremia was reported in the boosted PI monotherapy arm in some studies including MONOI trial, and that could have an impact on HIV-1 DNA reservoirs [1], [2], [3], [4], [7]. So it was interesting to compare evolution of HIV-1 plasma DNA viral load between DRV/r monotherapy arm and a DRV/r based triple therapy.

Our results showed that DRV/r monotherapy may have the same impact on reservoirs as a triple regimen in experienced patients after 2 years of treatment. Indeed there was no difference of delta HIV-1 DNA viral load (D0–W96) between the two arms and the observed variations were closed to previously published range [15]. Few studies have already addressed the HIV-1 DNA decrease in chronically infected patients receiving standard triple antiretroviral therapies [8], [9]. The MONARK data indicated that a lopinavir/ritonavir monotherapy regimen may have the same impact on reservoirs as a triple regimen in antiretroviral-naive patients after 1 year of treatment [10].

In MONOI trial, baseline HIV-1 DNA was identified as independent predictive factors of virological response in monotherapy arm [11]. This was in keeping with previous observations reporting that the baseline HIV-1 DNA level is associated with virological response on HAART [16], [17], [18] and in MONARK-study of induction lopinavir/ritonavir monotherapy [10]. A high baseline HIV-1 DNA level thus increased the risk of persistent low-level viremia during antiretroviral treatment [17]. Here we showed that baseline HIV-1 DNA was associated with CD4 nadir, pre therapeutic HIV-1 RNA viral load and residual viremia. Another study showed that whatever the duration of treatment HIV-1 DNA level was related with CD4 cell count nadir and plasma HIV-1 RNA zenith [14]. More recently, Chun et al demonstrated that residual plasma viremia correlated with the size of cellular HIV DNA reservoirs in infected patients receiving effective ARV therapy [19]. One limit of our study would be that quantification of total HIV-1 DNA in whole blood is not sensitive to measure the new event of cell infection during the intermittent viremia episodes. In conclusion, despite a higher proportion of intermittent viremia observed in some studies evaluating PI monotherapy strategy, we showed in MONOI trial than this does not seem to affect HIV-1 DNA level.
